# Assessment of Per- and Poly-Fluoroalkyl Substances (PFAS) and Polybrominated Diphenyl Ethers (PBDEs) in Surface Waters Used for Urban Water Supply in Brazil

**DOI:** 10.3390/toxics14020148

**Published:** 2026-02-02

**Authors:** Juliana de Souza-Araujo, Isadhora Camargo dos Santos, Hansel David Burgos Melo, Leila Soledade Lemos, Natalia Quinete, André Henrique Rosa

**Affiliations:** 1 Institute of Science and Technology, São Paulo State University (UNESP), Av. Três de Março, 511, Alto da Boa Vista, Sorocaba 18087-180, SP, Brazilandre.rosa@unesp.br (A.H.R.); 2 Institute of Environment, Department of Chemistry and Biochemistry, Florida International University, Biscayne Bay Campus, 3000 NE 151st Street, North Miami, FL 33181, USA

**Keywords:** PFAS, PBDEs, surface water, spatial distribution, seasonal variability

## Abstract

This study assesses the presence and distribution of per- and polyfluoroalkyl substances (PFAS) and polybrominated diphenyl ethers (PBDEs) in the surface waters of the Itupararanga Reservoir and the Sorocaba River, Brazil. Samples collected during the dry and rainy seasons were analyzed to determine their composition, spatial distribution, and seasonal variability. Results indicate the ubiquitous presence of PFAS, with significantly higher concentrations in the dry season, suggesting point sources of contamination, such as industrial and domestic discharges. Perfluorobutanoic acid (PFBA), Perfluorooctane sulfonate (PFOS), and Perfluorooctanoic acid (PFOA) were the predominant compounds, while 6:2 Fluorotelomer sulfonate (6-2FTS) stood out for its abundance in areas with industrial activity. For PBDEs, marked seasonal variability was observed, with higher concentrations during the rainy season, suggesting the mobilization of these compounds by surface runoff. BDE-209 was the most abundant congener, representing over 58% of the total concentration of PBDEs detected. Concentrations of PFAS and PBDEs in the study area are comparable to those reported globally, although there are differences associated with industrial practices and local environmental dynamics. The increased presence of short-chain PFAS and Deca-BDEs highlights the need for ongoing monitoring and the implementation of regulatory measures to mitigate contamination in water sources used for human consumption.

## 1. Introduction

Per- and polyfluoroalkyl substances (PFAS) comprise a large and diverse group of more than 4700 synthetic chemicals widely used in domestic, institutional, and industrial applications, including water- and grease-resistant coatings, food packaging, firefighting foams, surfactants, surface treatments, and insecticides [1,2]. Similarly, brominated flame retardants (BFRs), such as polybrominated diphenyl ethers (PBDEs), have been extensively applied to reduce flammability in plastics, textiles, electronic devices, and construction materials [3,4]. Due to their exceptional resistance to physical, chemical, and biological degradation, both PFAS and PBDEs are highly persistent in the environment and are classified as persistent organic pollutants (POPs), raising global concern regarding their long-term environmental fate and potential risks to human health [5].

Human exposure to PFAS and PBDEs has been associated with a wide range of adverse health outcomes, including endocrine disruption, immunotoxicity, hepatotoxicity, and carcinogenic effects [6,7,8,9]. Epidemiological and experimental studies have linked PFAS exposure to developmental effects, impaired immune responses, and thyroid hormone dysregulation [6,7,8,9], while PBDE exposure has been associated with neurodevelopmental toxicity and endocrine disturbances, particularly following prenatal exposure [10,11,12,13,14]. Owing to their physicochemical properties, PBDEs readily accumulate in biological tissues, including adipose tissue and breast milk [15,16], whereas PFAS exhibit high environmental mobility, resulting in widespread human exposure through multiple pathways.

Surface waters play a central role in the environmental distribution of PFAS and PBDEs, acting both as sinks for these contaminants and as direct exposure pathways in regions where rivers and reservoirs are used for public water supply. Major inputs to surface waters include industrial and domestic effluents, wastewater discharges, atmospheric deposition, and runoff from urbanized areas and landfills [17,18,19,20,21,22]. Consequently, contamination of surface waters by PFAS and PBDEs represents a relevant concern for drinking water safety, particularly in highly urbanized watersheds.

In Brazil, despite the large number of PFAS listed in international chemical inventories, documented uses have historically involved PFOS-related compounds, mainly associated with sulfluramid-based insecticides and metal plating applications [23,24]. Trade data indicate substantial past importation and production of PFOS precursors, suggesting potentially significant environmental releases [23,24]. Nevertheless, peer-reviewed information on the occurrence of PFAS in Brazilian surface waters remains scarce, with only a limited number of studies reporting their presence and frequently detecting PFOS, PFOA, and related compounds [25,26,27,28,29,30,31]. Similarly, although official data on PBDE importation and consumption are lacking, previous studies have reported their presence in several environmental matrices and biota in Brazil, indicating diffuse and persistent contamination sources related to urban activities and the disposal of consumer products [32,33,34,35,36,37,38,39,40,41,42,43,44,45].

Given this context, urban water supply systems in Brazil represent a critical yet understudied interface between environmental contamination and potential human exposure. In this study, the objectives were to (i) assess the occurrence and concentration levels of PFAS and PBDEs in surface waters used for public supply, (ii) evaluate their spatial distribution along a reservoir–river system influenced by urban and industrial activities, and (iii) investigate seasonal variations between dry and wet periods to infer dominant transport and source mechanisms. To address these objectives, surface water samples were collected from the Itupararanga Reservoir and the downstream Sorocaba River, an important urban water supply system in southeastern Brazil.

## 2. Materials and Methods

### 2.1. Chemicals

All standards were acquired from Wellington Laboratories (Guelph, ON, Canada). A 30-PFAS native standard mixture (PFAC30PAR, 1 μg mL^−1^ in methanol), a 19-PFAS isotopically mass-labeled standard mixture (MPFAC-24ES 1 μg mL^−1^ in methanol), and a labeled HFPO-DA standard solution (50 μg mL^−1^ in methanol) were used. Individual native standards of PBDEs were BDE-28, 47, 99, 100, 153, 154, 183 at 50 μg mL^−1^ in toluene and BDE-209 at 50 μg mL^−1^ in nonane. The internal standards were 3,30,4,40-tetrabromodiphenyl ether (BDE-77), 1,2,3-Tribromo-4-(2,3,4-tribromophenoxy)-benzene (BDE-128) at 50 μg mL−1 in nonane and decabromo [13C12]diphenyl ether (MBDE-209) at 25 μg mL^−1^ in toluene, and a recovery standard (2,2′,4,4′,6-Pentabromo [13C12]diphenyl ether (MBDE-100) at 50 μg mL^−1^ in nonane. The PBDE standards were weighed and dissolved in toluene, while the PFAS standards were prepared in methanol. Oasis-WAX cartridges 30 μm (6 mL, 150 mg, Waters, Milford, MA, USA) and Oasis-HLB cartridges (6 mL, 200 mg, Waters) were purchased from Merck KGaA (Darmstadt, Germany). The list of 30 PFAS and eight PBDEs with their respective internal standards applied for quantitation is included in Tables S1 and S2.

### 2.2. Sample Collection

**Figure 1:**
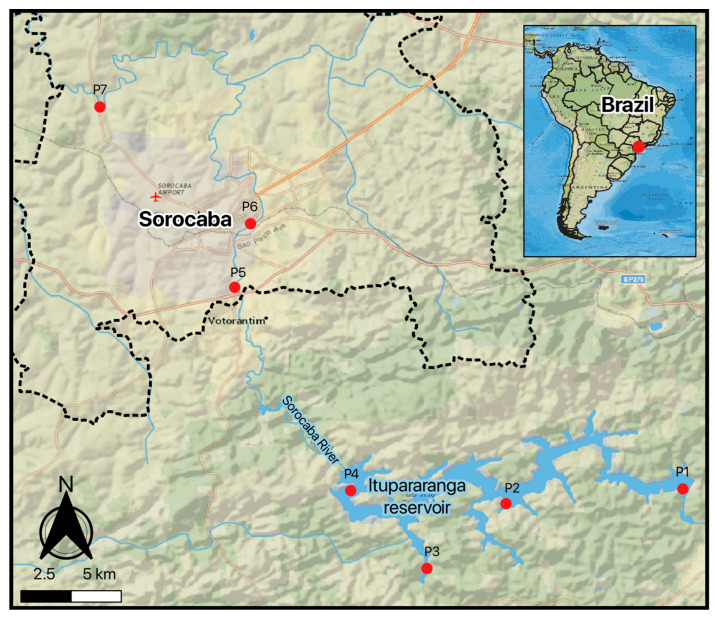
Study area in the Itupararanga Reservoir and Sorocaba River, southeastern Brazil, showing sampling points P1–P7. Sampling points were ordered from upstream (P1) to downstream (P7), following the direction of flow from the Itupararanga Reservoir to the Sorocaba River. Geographic coordinates can be found in Table S3.

In this study, sampling was conducted in the Sorocaba River and the Itupararanga Reservoir (Figure 1). The Sorocaba River is one of the most important rivers in the State of São Paulo and is the main tributary on the left bank of the Tietê River, with 227 km of extension. It is formed by the Sorocabuçu and Sorocamirim rivers and joins other small springs until their first impoundment, forming the Itupararanga Reservoir. The Itupararanga Reservoir has a drainage area of 936.51 km^2^, with 26 km of main channel. In addition, along with the Sorocaba River, the reservoir is intended to supply approximately 1.2 million people, as well as industries and agriculture in several cities, after conventional water treatment. The standard processes commonly applied in Brazilian drinking water treatment plants consist of coagulation, flocculation, sedimentation, filtration, and chlorination. While these treatments are effective for the removal of particulate matter and pathogens, they are not designed to remove highly persistent and mobile organic contaminants such as PFAS and PBDEs, which may therefore remain in treated water supplied to the population. The watercourses also pass through extensive areas of vegetable cultivation, pastures, condominiums, farms, and summer houses, all of which contribute to the influx of nutrients and toxic substances into the reservoir, potentially making the limnological conditions unstable [46].

All the samples were taken in two sampling events during the dry (October 2022) and wet (February 2023) seasons, respectively. According to meteorological data from the CBH-SMT (Sorocaba and Middle Tietê Hydrographic Basin Committee) reports, the inflow rate (water inflow) during the 2022 dry season was very low, at 2.71 m^3^/s, compared to the expected average of 8.08 m^3^/s for the period. On the other hand, data from the beginning of the 2023 flood season indicated an inflow rate of 20.28 m^3^/s, while the expected inflow rate was 19.88 m^3^/s. Sampling points were arranged from upstream (P1–P4) within the Itupararanga Reservoir to downstream locations (P5–P7) along the Sorocaba River, following the natural direction of water flow. The seven sampling points were selected to represent a spatial gradient along the Itupararanga Reservoir and the downstream Sorocaba River, encompassing areas with distinct hydrological characteristics and land use patterns. Points located within the reservoir were influenced by different activities along the margins, including tourism-related developments (e.g., hotels), agricultural areas, and the reservoir dam, which affects water residence time and dilution processes (Table S3). Downstream points along the Sorocaba River were located in the urban area of Sorocaba city, where higher population density, wastewater discharges, urban runoff, and industrial activities may influence contaminant concentrations. A qualitative summary of the main hydrological and land use characteristics of each sampling site is provided in Table S3.

Samples were collected in 500 mL pre-cleaned high-density polyethylene (HDPE) bottles stored on ice during transportation and then refrigerated at 4 °C until the analysis. The samples were refrigerated for less than a week before analysis. Three surface water samples were collected from each site, selected based on land use characteristics and occupation of the banks, to evaluate the impact of anthropogenic activities on the reservoir’s water quality. A total of 7 sampling points were assessed, and six bottles of water (three for PBDEs and three for PFAS) were collected at each site, encompassing a total of 21 surface water samples. Mean concentrations and standard deviations reported in Table 1 and Table 2 represent biological variability among three independent surface water samples collected at each site.

### 2.3. Sample Preparation and Extraction

All materials used during sample handling and extraction, including containers, bottles, and tubing, were thoroughly cleaned prior to use. The cleaning procedure consisted of sequential rinses with ultrapure water, acetone, hexane, and methanol, each applied at least twice. After cleaning, materials were allowed to air-dry to ensure complete solvent evaporation.

PFAS were extracted from surface water samples using solid-phase extraction (SPE) based on a previously established method [47], with minor adjustments. Briefly, 500 mL of unfiltered water samples were fortified with 200 μL of an internal standard solution prepared in methanol, containing a mixture of 20 PFAS at a concentration of 2.5 ng/mL. Samples were then loaded onto weak anion exchange (WAX) SPE cartridges that had been sequentially conditioned with 6 mL of methanol containing 0.1% ammonium hydroxide, followed by 6 mL of methanol and 6 mL of Milli-Q water. After sample loading, the cartridges were dried under vacuum for approximately 40 min to remove residual water. Target compounds were subsequently eluted using 10 mL of methanol containing 0.1% ammonium hydroxide. The resulting extracts were concentrated to dryness under a gentle stream of nitrogen in a water bath maintained at 60 °C. Dried extracts were reconstituted to a final volume of 1 mL using a solution of 5 mM ammonium acetate and methanol (90:10, *v*/*v*) prior to instrumental analysis.

PBDEs were extracted using SPE following an established protocol [48] with slight modifications. For this procedure, 500 mL of unfiltered water samples were spiked with isotopically labeled internal standards, including 10 μL of BDE-77 and BDE-128 (4 ng/μL) and 20 μL of MBDE-209 (5 ng/μL). Oasis HLB cartridges (Waters) were conditioned sequentially with 15 mL of hexane, 15 mL of dichloromethane, 15 mL of methanol, and 15 mL of Milli-Q water prior to sample loading. Following sample percolation, cartridges were dried under vacuum, and analytes were eluted using a mixture of dichloromethane/hexane (1:1, *v*/*v*) followed by dichloromethane/acetone (1:1, *v*/*v*). The combined eluates were concentrated under a nitrogen stream to near dryness and reconstituted in 100 μL of hexane. Subsequently, 10 μL of recovery standard (MBDE-100 at 1 ng/μL) was added, and the volume was adjusted with hexane to a final volume of 200 μL for chromatographic analysis. The vials were kept in the freezer until the analysis by GC-MS for PBDEs and refrigerated until the LC-MS/MS analysis for PFAS.

### 2.4. Instrumental Conditions

Quantitative analysis of PFAS was carried out at Florida International University (FIU) using liquid chromatography coupled to tandem mass spectrometry (LC–MS/MS), following an established analytical approach with minor adaptations. Aliquots of 100 μL of the pre-concentrated extracts were injected into an Agilent 1290 Infinity II liquid chromatograph (Agilent Technologies, Santa Clara, CA, USA) coupled to an Agilent 6470 triple quadrupole mass spectrometer (Agilent Technologies), equipped with an Agilent Jet Stream electrospray ionization (AJS-ESI) source (Agilent Technologies). To minimize background contamination, the LC system was fitted with PFAS-free tubing. In addition, a delay column (Hypersil GOLD aQ C18, 20 × 2.1 mm, 12 μm; Thermo Fisher Scientific, Waltham, MA, USA) was installed between the mobile phase mixer and the autosampler injector to further reduce potential PFAS interference originating from the solvent delivery system. Chromatographic separation of PFAS congeners was achieved using a Hypersil GOLD perfluorinated phenyl (PFP) analytical column (150 mm × 2.1 mm, 3 μm; Thermo Fisher Scientific), protected by a PFP guard column (Hypersil GOLD PFP, 5 μm drop-in guard cartridges; Thermo Fisher Scientific), maintained at 50 °C. The mobile phases consisted of 5 mM ammonium formate in water and methanol, delivered at a constant flow rate of 0.4 mL min^−1^. Gradient elution conditions, as well as optimized mass spectrometric parameters, are provided in Tables S4 and S5, respectively. Data acquisition was performed in negative ionization mode using multiple reaction monitoring (MRM) for the simultaneous detection and quantification of individual PFAS compounds, with monitored transitions listed in Table S6.

PBDEs were analyzed by gas chromatography coupled to electron ionization mass spectrometry (GC–EI–MS) using an Agilent 7890A gas chromatograph interfaced with a 5975C quadrupole mass spectrometer (Inert MSD with Triple-Axis HED-EM detector; Agilent Technologies). Chromatographic separation was achieved using a DB-5MS capillary column (15 m length × 0.25 mm internal diameter × 0.25 μm film thickness; J&W Scientific, Folsom, CA, USA). The oven temperature program started at 80 °C, held for 2 min, followed by an increase to 170 °C at a rate of 20 °C min^−1^ with a hold of 5.5 min, and a final ramp to 320 °C at 25 °C min^−1^, maintained for 10 min, resulting in a total run time of 28 min. Helium (99.999% purity) was used as the carrier gas under constant flow conditions at 1.8 mL min^−1^. Samples and standards (1 μL) were introduced into the system in splitless mode, with a splitless time of 1.5 min. Injector, quadrupole, and transfer line temperatures were set at 320 °C, 300 °C, and 300 °C, respectively. Mass spectrometric detection was performed in electron ionization mode at 70 eV. Data acquisition was carried out using time-scheduled selected ion monitoring (SIM), targeting the two most intense or selective fragment ions for each analyte. The monitored ions are listed in Table S7.

### 2.5. Quality Control

All samples were processed using procedures that have been previously validated [47,48,49]. For PBDEs, a 5-point calibration curve was performed from the BDEs native standard solution (20 to 1000 pg μL^−1^ and BDE-209 750 to 2500 pg μL^−1^). For PFAS, an 11-point calibration was plotted with concentrations ranging from 2 to 1000 ng L^−1^ using diluted solutions from the 30-PFAS native standard working solution mixture. The method detection limit (MDL) and quantification (MQL) were estimated based on a signal-to-noise ratio (S/N) of 3:1 and 10:1, respectively. For PFAS, MDLs have been previously statistically determined by multiplying the standard deviation of eight replicate spiked sample concentrations by the one-sided Student’s t value at the 99% confidence level, as described in [47] (Tables S8 and S9).

### 2.6. Statistical Analysis

Descriptive statistics were done using Excel for Mac (v.16.16.22). To assess differences in concentrations across sites and between seasons, a univariate permutational multivariate analysis of variance (PERMANOVA) based on Euclidean distance matrices and 9999 permutations was performed using the ‘adonis’ function from the vegan package in R Studio (version 1.2.5019). The plots were generated with the ggplot2 package.

## 3. Results

### 3.1. Occurrence and Concentrations of PFAS in Itupararanga Reservoir and in the Sorocaba River

**Figure 2:**
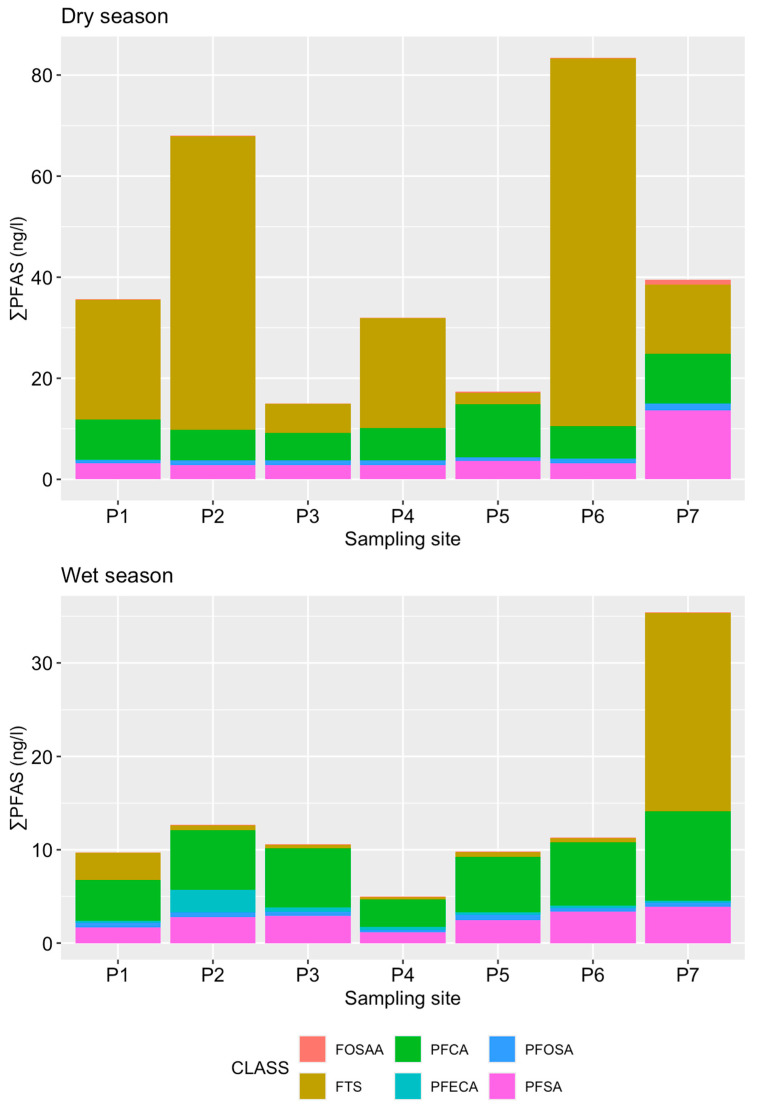
Spatial distribution of ∑30PFAS class in surface water samples collected during the dry and wet (flood) seasons in the metropolitan region of Sorocaba.

Among the 30 PFAS analyzed, 29 congeners were detected at one or more sites in the water samples, with ADONA (also known as 9Cl-PF3ONS) below the MLD at all sites. Among the PFAS, PFBA, PFPeA, PFBS, PFHxA, PFPeS, PFHxS, PFHpA, 6-2 FTS, FBSA, PFOA, PFOS, PFNA, PFDA, and FOSA were detected in all samples analyzed. In addition, PFDoA, PFTrDA, 4-2 FTS, FHxSA, and PFTeDA were detected in more than 80% of the samples. The mean concentrations obtained from triplicates of each compound are shown in Table 1, and the seasonal and spatial distribution of PFAS are shown in Figure 2. The sum of PFAS concentration (∑30PFAS) in surface water samples showed a highly significant seasonal variation (Pseudo-F = 18.87, *p* < 0.001), ranging from 108.07 ± 9.77 to 256.42 ± 29.19 ng/L during dry season and from 46.6 ± 2.8 to 211.79 ± 31.34 ng/L during wet season, in agreement with several studies reviewed by Podder et al. [50], where PFAS concentrations in surface water decrease by almost 50% in the wet season, presumably due to dilution effects associated with increased water inflow and the high solubility and mobility of these compounds.

During the dry season, the highest concentration was from 6 to 2 FTS (average: 50.97 ng/L), followed by PFBA (average: 47.39 ng/L), PFOS (average: 19.82 ng/L), and PFPeA (average: 14.20 ng/L), while in the wet season, the highest average concentration was from PFBA (38.46 ng/L), followed by PFOS (13.34 ng/L), 6-2 FTS (11.65 ng/L), and PFOA (10.25 ng/L) (Figure 2).

Among the compounds detected, PFHxA, PFOA, PFBS, and PFOS are regarded as the most frequently occurring PFAS globally [50]. In fact, most studies carried out over the last few decades in Brazil reported PFOS and PFOA among the most abundant compounds in surface water samples, even after restrictions and regulatory measures have been implemented to limit their use and production. For example, Quinete et al. [25] conducted the first analyses in Guanabara Bay and Paraiba do Sul River in the state of Rio de Janeiro and found PFOA and PFOS at concentrations in the ranges of 0.7–3.25 and 0.4–0.92 ng/L, respectively. More recently, Stefano et al. [29] reported concentrations ranging from 4.4 to 12.8 ng/L for PFOA and 3.5 to 6.6 ng/L for PFOS in surface waters from the northern industrial region of the city of Porto Alegre in Rio Grande do Sul. The authors concluded that PFAS concentrations in those zones (subject to discharges from the leather industry, urban sewage, and industrial waste) were higher than in areas with predominantly agricultural activities. Similarly, recent findings from Starling et al. [31] from Pampulha Lake in Belo Horizonte, a UNESCO World Heritage Site, revealed PFOA concentrations ranging from 973.1 to 45,489.9 ng/L, which are higher than those previously reported in other Brazilian surface waters. The authors noted that the study area is surrounded by manufacturing plants from the chemical, food and beverage, and textile sectors.

The predominance of PFBA, which is often used in the manufacture of products that require resistance to stains, water, and oil, such as food packaging, textiles, and non-stick cookware, is believed to be the result of point sources, especially due to the discharge of untreated municipal and industrial wastewater. Meanwhile, the abundance of 6:2 FTS, used as a surfactant in firefighting foams, as well as in the electroplating industry as a mist suppressant in chrome baths, may be related to the fact that at least 11 electroplating industries are present in the study area. Also, the higher prevalence of shorter chain compounds compared to PFOS and PFOA concentrations could be attributed to global efforts over the past decades to limit the use of long-chain PFAS, increased use practices for short-chain PFAS in the industry, or the degradation of PFAS precursors leading to terminal perfluoroalkyl acids [50,51].

There was a significant difference in total PFAS concentrations between collection sites in both seasons (dry: Pseudo-F = 10.62, *p* < 0.001; wet: Pseudo-F = 32.45, *p* < 0.001), reflecting distinct hydrological conditions and land-use characteristics associated with each sampling point. Among the sites, point P7 stood out with higher concentrations than other sampling points in both seasons (dry: 216.52 ± 5.85 ng/L; wet: 211.79 ± 31.34 ng/L), indicating a major contribution of PFAS containing-effluent releases along the Sorocaba River. This spatial pattern is supported by statistically significant differences among sampling sites (PERMANOVA, *p* < 0.001) and indicates cumulative contributions from urban wastewater discharges, surface runoff, and reduced dilution capacity as the river flows through densely urbanized areas of Votorantim and Sorocaba. The sites P5 and P6 showed the highest PFAS levels after P7, further reflecting the increasing influence of urbanization along the river continuum. Overall, these results highlight the strong control exerted by hydrological connectivity and anthropogenic pressures on PFAS distribution in surface waters used for public supply.

Historically, the Sorocaba River has faced high levels of pollution due to industrial activities, mining, and the discharge of untreated sewage. However, since the 1980s, there has been a significant effort to restore the river’s environment, including the implementation of sewage treatment plants and decontamination programs. Despite significant progress in sewage treatment, pollution sources continue to impact the water quality of the Sorocaba River. Each year, local media report diffuse pollution events from urban, industrial, and agricultural areas, especially during rainy periods, when runoff carries waste into the river. In addition, irregular dumping also persists in some regions, such as the East Zone of Sorocaba city, where sewage is reportedly discharged directly into streams feeding the river. Alongside previous studies, these findings emphasize that in Brazil, highly urbanized and industrialized areas with inadequate sanitation infrastructure face notable PFAS contamination in surface waters.

The PFAS concentrations at all points of the Itupararanga Reservoir were much lower than those found in similar environments in Brazil, such as the Pampulha lagoon (191–12,400 ng/L) [6], the Três Marias reservoir (36,880–46,020 ng/L) [30]. In contrast, PFAS concentrations in the Sorocaba River were higher when compared to other regions worldwide, such as the Indus River in Pakistan (19–114 ng/L) [52], the Rhine River (4.08–38.5 ng/L) and the Ruhr River (64.8–96.7 ng/L) [53], the Seine River (31–91 ng/L) [54] and the Elbe River (7.6–26.4 ng/L) [55] in Europe, and to the Altamaha River in North America (5.7–6.3 ng/L) [56].

During the dry season, PFAS concentrations were higher at points close to the headwaters of the Itupararanga Reservoir (P2: 256.42 ± 29.19 ng/L and P1: 194.41 ± 1.99 ng/L) compared to other locations, probably due to the release of domestic effluents from a resort on the reservoir’s banks at point P2 and from the Sorocamirim and Sorocabuçu rivers, which flows through municipalities upstream of the reservoir at point P1. Along the reservoir, PFAS concentrations decrease, possibly due to dilution effects or interactions of pollutants with reservoir sediments. The spatial variation of individual compounds shows a very similar pattern at both sampled locations.

**Figure 3:**
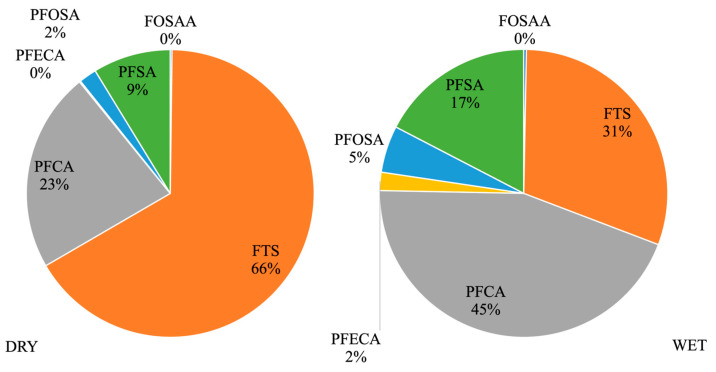
Percentage of PFAS composition categorized by class during the dry and wet periods.

Given the variation in chemical composition from sample to sample, we have explored PFAS distribution categorized by class: perfluoroalkyl carboxylic acids (PFCA: PFBA, PFDA, PFDoA, PFHpA, PFHxA, PFNA, PFOA, PFOUDS, PFPeA, PFTeDA, PFTrDA, and PFUdA), perfluoroalkyl sulfonic acids (PFSA: PFBS, PFDS, PFHpS, PFHxS, PFNS, PFONS, PFOS, and PFPeS), fluorotelomer sulfonic acids (FTS: 4-2 FTS, 6-2FTS, and 8-2 FTS), perfluoroalkyl ether carboxylic acids (PFECA: Adona and GenX), perfluoroalkane sulfonamido acetic acids (FOSAA: N-EtFOSAA and N-MeFOSAA) and perfluoroalkane sulfonamides (PFOSA: FBSA, FHxSA, and FOSA). In all samples, when combining the concentrations of all compounds belonging to the same category, it is observed that FTS and PFCA represent 76% to 89% of the total PFAS detected. During the dry season, the predominant classes found were FTS > PFCA > PFSA > PFOSA, whereas in the wet season, it was PFCA > FTS > PFSA > PFOSA (Figure 3).

In agreement with our findings, Ackerman Grunfeld et al. [51] analyzed sources of PFAS in global surface water and groundwater, categorizing source products into two groups: related (for example, firefighting training area) and not related (for example, production facilities using or producing PFAS, landfills) to aqueous film-forming foam (AFFF). They examined 943 non-AFFF consumer products, distributed across 15 categories and from 38 studies, identifying 113 PFAS compounds. Despite the challenges in comparing PFAS classes between studies, FTS and PFCAs predominated, representing medians of 72% and 25% of the total PFAS subclass mass in most of the product categories investigated (for example, coatings, cosmetics, and textiles), respectively. The authors concluded that non-AFFF sources were considered the main contributors to contamination. Based on this information and the composition of FTS and PFCAs found in our study, potential sources are likely not AFFF-related and instead could be linked to fluorinated cleaning agents and waxes, electroplating facilities, and degradation of precursor compounds in consumer products such as cosmetics and household items.

### 3.2. Occurrence and Concentrations of PBDEs in the Sorocaba River

**Figure 4:**
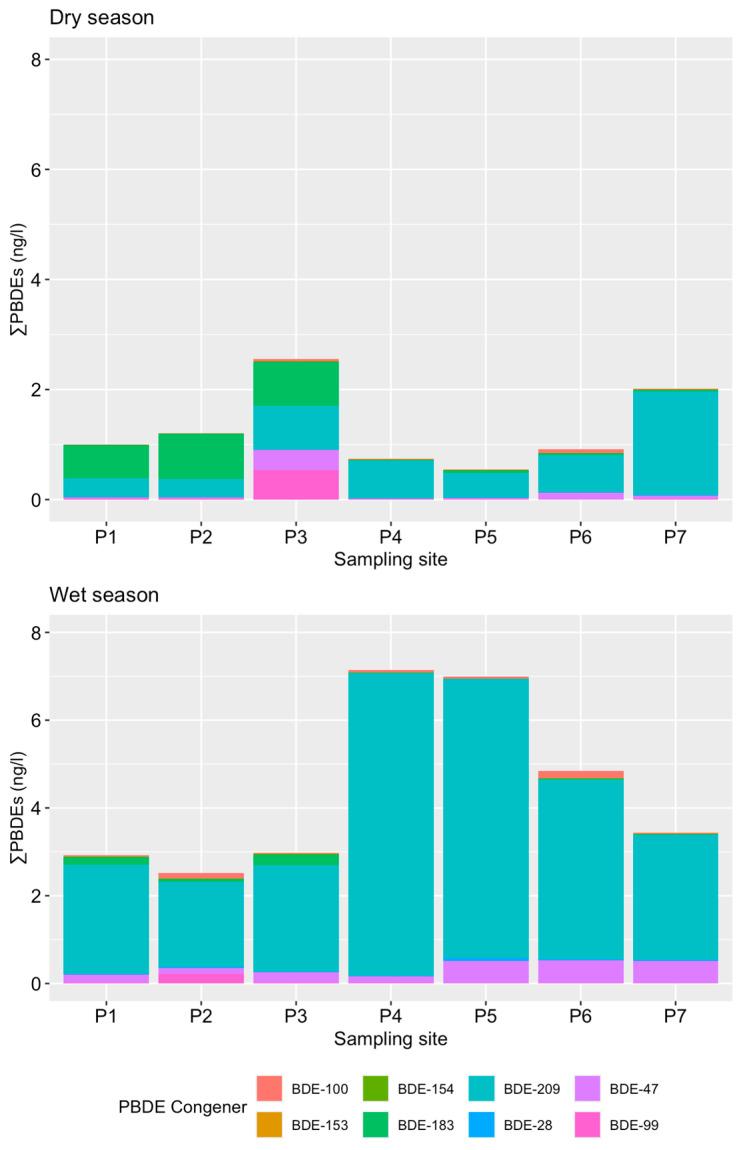
Spatial distribution of total PBDEs in surface water samples collected during the dry and wet (flood) seasons in the metropolitan region of Sorocaba.

Regarding PBDEs, all analyzed congeners were detected in the samples, with average concentrations shown in Table 2, and the seasonal and spatial distribution among the sampled sites illustrated in Figure 4. BDE-209, BDE-47, BDE-99, and BDE-100 were detected in all samples, while BDE-183 was found in over 80% of the samples. The other congeners were detected in quantifiable concentrations in ≤50% of the samples. Concentrations ranged from 0.55 to 2.56 ng/L in the dry season and from 2.51 to 7.15 ng/L in the wet season. PBDE contamination in water sources around the world is quite common, and the values found in Brazil are similar to those in studies in China (not detected to 7.87 ng/L with an average value of 2.59 ng/L [57], 0.49 to 17.4 ng/L with an average value of 1.38 ng/L [58]), Taiwan (0.03 to 1.02 ng/L [59]), and South Africa (2.60 to 4.83 ng/L [60]). However, they are higher than values found in England (0.009 to 0.17 ng/L [61]), North America (0.0002 to 0.006 ng/L [62]), and Nigeria (0.03 to 0.54 ng/L reported by [63]; 0.02 to 0.36 ng/L [64]), likely due to stricter control and regulations that exist in Europe and North America.

Similar to PFAS, a significant seasonal variation in PBDE concentrations was observed (Pseudo-F = 46.12, *p* < 0.001); however, the wet season was the one exhibiting the highest total PBDE concentrations and may be related to enhanced surface runoff and mobilization of particle-associated contaminants, as reported in the previous studies discussed below. Although no direct measurements of suspended solids or particulate organic matter were available in this study, this interpretation is presented as a plausible mechanism based on the well-documented hydrophobic nature and sediment affinity of PBDEs. The highest concentration among all PBDE congeners during the dry season was BDE-209 (up to 1.89 ng/L, mean: 0.74 ng/L), followed by BDE-183 (mean: 0.33 ng/L), BDE-47 (mean: 0.09 ng/L), and BDE-99 (mean: 0.09 ng/L). During the wet season, the highest mean concentration among PBDE congeners was also BDE-209 (up to 6.90 ng/L, mean: 3.86 ng/L), followed by BDE-47 (0.33 ng/L), BDE-183 (0.07 ng/L), and BDE-100 (0.07 ng/L) (Table 2; Figure 4). In addition, there was a significant difference in the total PBDE concentrations between the sampling sites in both seasons (dry: Pseudo-F = 59.77, *p* < 0.001; wet: Pseudo-F = 17.88, *p* < 0.001), reflecting the influence of local land use and hydrological conditions along the system. The highest values were observed at point P3 during the dry season (2.56 ng/L), located in front of a residential building within the reservoir area, suggesting localized inputs associated with urban infrastructure and household sources. In contrast, lower concentrations at other reservoir sites indicate limited PBDE mobility under low-flow conditions.

During the wet season, PBDE concentrations increased markedly at sites P4 (7.15 ng/L) and P5 (6.99 ng/L) during the wet season, located near the reservoir dam and upstream of Sorocaba city, respectively, as well as at P6 (4.84 ng/L) within the urban center. These sites are characterized by proximity to highways, urbanized areas, and hydraulic structures, which may enhance the accumulation and mobilization of particle-associated contaminants during periods of increased runoff. This seasonal shift in spatial patterns is consistent with the hydrophobic nature of PBDEs and their affinity for particulate matter, highlighting the role of land use and hydrological dynamics in controlling PBDE distribution. There is a very similar pattern of PBDE composition at all sampled sites (Figure 4).

Corroborating our findings, a recent review on PBDEs in the affected environmental systems indicated that PBDEs are lower in the dry season than in the wet season, with BDE 209 being the predominant congener [65]. For example, Olutona et al. [63] assessed the concentrations of Σ6PBDEs in stream water from Asunle stream in Nigeria and found higher levels during the wet season (0.18–0.45 ng/L), potentially linked to active mobilization of leached PBDE congeners by erosion into the water body. Similarly, Tongu et al. [64] determined the concentrations of Σ6PBDEs in water samples from open city drains in the Makurdi Metropolitan Area, North Central Nigeria, and also found higher levels of Σ6PBDEs during the wet season, with values ranging from 0.05 to 0.28 ng/L in the dry season and 0.02 to 0.36 ng/L in the wet season. In China, Pei et al. [58] investigated the spatial and seasonal distributions of Σ14PBDEs in water along the Yellow River and found lower PBDE concentrations in samples collected during the dry season (median of 1.38 ng/L) compared to those in samples collected during the wet season (median of 7.30 ng/L). All authors agree that the presence of adjacent industrial and urbanized regions contributes to the input of PBDEs into the studied water bodies.

**Figure 5:**
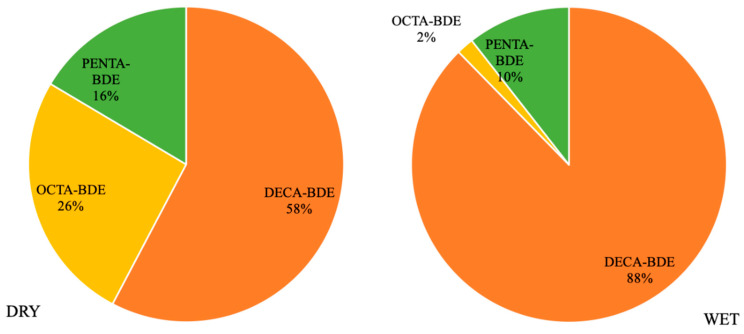
Percentage of PBDE composition categorized by class during the dry and wet periods.

The pollutants were categorized by class into Penta-BDEs (BDE-28, -47, -99, and -100), Octa-BDEs (BDE-153, -154, and -183), and Deca-BDEs (BDE-209). In all the analyzed samples, Deca-BDEs alone represented 58 to 88% of the total PBDEs detected, even though this class is represented by the minority of the compounds analyzed (Figure 5). This result is similar to findings from municipal and industrial wastewater treatment plants in Ulsan city, Korea [66], and in the Yellow River [58] and Jiaozhou Bay [67] in China. Since Deca-BDEs are widely used as flame retardants in plastics, electronics, and construction materials, their greater abundance in the environment may be expected. Additionally, this class has high molecular mass and low solubility, tending to accumulate in sediments and being more resistant to photodegradation and biodegradation processes [68]. Recent studies conducted in Brazil in 2024 reinforce the predominance of Deca-BDE, detecting significant amounts of this compound in both household dust [69], recyclable materials [45], and wastes from construction and demolition, soft furnishings, and end-of-life vehicles [70]. This ubiquitous presence suggests that products containing Deca-BDE, such as furniture, electronics, and plastics, are important sources of emissions, including releases to natural water sources. On the other hand, the second most abundant class is octa-BDEs, particularly BDE-183. Previous research has identified this compound as one of the main contributors to the electronic waste (e-waste) generated in Brazil [45] and to household dust in Brazilian homes [69]. In this sense, the significant concentrations of BDE-183 in the waters of Sorocaba could be related to its use in large quantities.

### 3.3. Current Regulations and Potential Risks of Persistent Pollutants on the Environment and Human Health

The detection of reasonably high concentrations of PFAS and PBDEs in the Sorocaba River and the Itupararanga Reservoir raises serious environmental and public health concerns. The Itupararanga Reservoir is a strategic source of water supply for five municipalities in the region, which makes the contamination of its waters a problem of great regional relevance. Despite advances in basic sanitation, with high rates of sewage collection and treatment, wastewater treatment plants in the region predominantly rely on conventional activated sludge processes. While effective for the removal of organic matter and nutrients, these systems are not designed to remove highly persistent organic pollutants such as PFAS and PBDEs, allowing these compounds to persist in treated effluents and surface waters.

The fact that the Sorocaba River still receives treated sewage and, in some places, raw sewage makes the river a constant source of contaminant recharge to the reservoir, especially during the rainy season, when surface runoff intensifies the pollutant load. This situation exemplifies the paradox faced by many urbanized regions in developing countries: although access to clean water has expanded, emerging contaminants for which there is no adequate removal technology continue to threaten water security.

In countries such as the United States, the Environmental Protection Agency (US EPA) has set extremely stringent limits for the presence of certain PFAS in drinking water. In April 2024, the USEPA established maximum contaminant limits (MCLs) for six PFAS in drinking water: 4 ng/L for compounds such as PFOA and PFOS and 10 ng/L for PFHxS, PFNA, and HFPO-DA (GenX), recognizing the toxicity of these contaminants even at extremely low concentrations [71]. In the present study, the concentrations of PFOA and PFOS in surface water samples—particularly from the Itupararanga Reservoir—exceeded these regulatory thresholds by several folds, reaching up to 12.71 ng/L for PFOA and 18 ng/L for PFOS. These findings underscore a significant environmental and public health concern, especially considering that the reservoir serves as an important resource for human consumption after conventional treatment. Similarly, in 2020, the European Union approved Regulation (EU) 2020/784 [72], which amends Annex I to Regulation (EU) 2019/1021 on persistent organic pollutants [73] and includes specific restrictions on PBDEs in products and waste, as well as limits on the presence of PFAS in food and proposes a progressive ban on these substances by 2030. The measure aims to gradually eliminate the use of these substances due to their environmental persistence and potential for bioaccumulation. Australia has developed the “PFAS National Environmental Management Plan” (NEMP), which provides clear national guidelines for the management, monitoring, and removal of PFAS in different environmental matrices [74]. The NEMP is an adaptive document, regularly updated to reflect new scientific evidence and guidance.

Meanwhile, Brazil still lacks specific regulations that establish maximum limits for PFAS in drinking water, sediments, or fish, and has very limited regulations on PBDEs, focused almost exclusively on hazardous solid waste. The lack of legal parameters for these emerging contaminants prevents effective corrective actions and makes it difficult to establish public policies for environmental protection and public health. This regulatory gap is particularly worrying given the growing evidence of the ubiquity and persistence of these compounds in Brazilian water bodies.

In the case of Itupararanga, the continued presence of PFAS and PBDEs represents a latent risk and reveals a critical gap between water supply management and contemporary environmental challenges, highlighting the urgent need for systematic monitoring programs and the formulation of preventive public policies to limit the release of these substances into the environment.

## 4. Conclusions

The results of this study revealed the significant presence of a wide range of PFAS and PBDEs in surface water samples collected from various points in the Itupararanga Reservoir and in the Sorocaba River in the state of São Paulo, Brazil. The predominant detection of compounds such as PFBA, PFOS, and PFOA in all samples, along with seasonal and spatial variability of PFAS concentrations, indicates point sources of contamination, such as domestic and industrial effluents, especially in the dry season. In addition, the detection of Deca-BDE in high concentrations, particularly during the rainy season, highlights the importance of local sources such as the use of flame retardants in construction materials, furniture, and electronics. Comparison with international studies suggests that the concentrations of PFAS and PBDEs found in this study are in line with patterns observed globally, though with some variations reflecting local industrial practices and the unique characteristics of the region’s aquatic ecosystems. The prevalence of short-chain PFAS compounds, such as PFBA and 6-2FTS, and the predominance of Deca-BDEs reinforce the need for continuous monitoring and evaluation of public policies to reduce the sources of emissions of these pollutants, which continue to impact water quality and aquatic ecosystems. In the global context, this study highlights the urgent need for Brazil to advance in the development of specific standards for PFAS and PBDEs, in alignment with international best practices. Immediate action is needed not only to monitor and control contamination but also to review urban planning, legislation on industrial effluents, and policies related to chemical safety, aiming to protect human health and the integrity of ecosystems.

## Figures and Tables

**Table 1:** Mean concentrations and standard deviations of PFAS in ng/L at the seven collection points sampled during the dry and wet periods.

Compound	DRY							WET						
	P1	P2	P3	P4	P5	P6	P7	P1	P2	P3	P4	P5	P6	P7
**4-2 FTS**	0.46 ± 0.07	1.6 ± 1.81	0.11 ± 0.05	0.48 ± 0.13	0.02 ± 0	2.78 ± 3.81	0.27 ± 0.03	0.05 ± 0.02	0.01 ± 0.02	0.05 ± 0	0.01 ± 0.01	0.01 ± 0.02	<MDL	0.3 ± 0.21
**6-2FTS**	69.82 ± 1.3	155.44 ± 18.86	16.79 ± 11.08	64.32 ± 15.55	6.53 ± 0.02	4.39 ± 0.22	39.46 ± 0.66	8.77 ± 0.04	1.55 ± 0.06	1.19 ± 0.05	0.8 ± 0.09	1.39 ± 0.04	1.41 ± 0.09	63.01 ± 39.16
**8-2 FTS**	0.68 ± 0.09	1.58 ± 1.07	0.22 ± 0.04	0.42 ± 0.07	0.4 ± 0.11	1.84 ± 2.42	1.19 ± 0.24	0.05 ± 0.01	<MDL	<MDL	<MDL	0.01 ± 0.01	0.02 ± 0.03	0.33 ± 0.2
**Adona**	<MDL	<MDL	<MDL	<MDL	<MDL	<MDL	<MDL	<MDL	<MDL	<MDL	<MDL	<MDL	<MDL	<MDL
**FBSA**	0.06 ± 0.03	0.03 ± 0	0.11 ± 0.06	0.16 ± 0.02	0.15 ± 0.11	0.14 ± 0.06	0.18 ± 0.09	0.05 ± 0.02	0.18 ± 0.07	0.27 ± 0.1	0.09 ± 0.06	0.26 ± 0.02	0.21 ± 0	0.41 ± 0.19
**FHxSA**	0.03 ± 0.04	<MDL	0.01 ± 0.01	0.01 ± 0.01	0.02 ± 0.01	<MDL	0.08 ± 0.08	0.01 ± 0.01	0.05 ± 0.07	0.04 ± 0.01	0.01 ± 0	0.02 ± 0	0.01 ± 0.01	0.14 ± 0.09
**FOSA**	2.03 ± 0.41	2.88 ± 0.82	2.56 ± 0.85	2.92 ± 1.41	2.01 ± 0.55	2.53 ± 0.04	3.58 ± 0.21	1.47 ± 0.09	1.18 ± 0.32	1.1 ± 0.32	0.8 ± 0.28	1.41 ± 0.27	0.99 ± 0.21	0.88 ± 0.16
**GenX**	0.1 ± 0.1	<MDL	<MDL	<MDL	<MDL	0.11 ± 0.16	0.13 ± 0.22	0.4 ± 0.15	4.81 ± 6.43	0.87 ± 0.53	0.66 ± 0.55	0.53 ± 0.2	0.54 ± 0.76	0.35 ± 0.36
**N-EtFOSAA**	0.07 ± 0.12	0.22 ± 0.38	0.06 ± 0.08	0.06 ± 0.03	0.23 ± 0.14	0.2 ± 0.17	0.67 ± 0.24	<MDL	<MDL	0.04 ± 0.03	0.03 ± 0.05	0.14 ± 0.08	0.15 ± 0.05	0.09 ± 0.08
**N-MeFOSAA**	0.07 ± 0.01	0.04 ± 0.07	0.05 ± 0.04	0.06 ± 0.06	0.24 ± 0.03	0.01 ± 0.01	1.26 ± 0.38	0.05 ± 0	0.03 ± 0.04	0.09 ± 0.01	<MDL	<MDL	<MDL	0.04 ± 0.04
**PFBA**	48.77 ± 0.49	39 ± 3.65	37.61 ± 1.89	44.53 ± 0.29	72.25 ± 62.4	44.68 ± 0.7	44.01 ± 1.29	34 ± 11.34	47.07 ± 0.63	48.77 ± 0.46	21.13 ± 0.87	41.06 ± 0.84	34.99 ± 0.93	41.05 ± 0.66
**PFBS**	4.55 ± 0.08	4.25 ± 0.17	4.59 ± 0.05	3.83 ± 0.14	4.76 ± 0.02	4.05 ± 0.03	9.20 ± 1.05	2.45 ± 1	4.68 ± 0.37	3.51 ± 0.1	2.05 ± 0.1	3.6 ± 0.01	4.78 ± 0.01	6.1 ± 0.3
**PFDA**	1.06 ± 0.03	1.15 ± 0.14	0.68 ± 0.07	0.73 ± 0.07	1.04 ± 0.1	0.76 ± 0.15	1.83 ± 0.23	0.43 ± 0.12	0.62 ± 0.02	0.38 ± 0	0.22 ± 0	0.39 ± 0	0.56 ± 0	1.03 ± 0.07
**PFDoA**	0.17 ± 0.01	0.84 ± 0.24	0.2 ± 0.14	0.13 ± 0.11	0.28 ± 0.05	0.23 ± 0.05	0.3 ± 0.02	0.04 ± 0.04	0.18 ± 0.15	0.11 ± 0.02	0.03 ± 0.04	0.3 ± 0.03	0.06 ± 0	0.14 ± 0.06
**PFDS**	0.01 ± 0.02	0.04 ± 0.05	0.57 ± 0.01	<MDL	<MDL	<MDL	<MDL	<MDL	<MDL	0.16 ± 0.28	0.08 ± 0.14	0.03 ± 0.05	0.91 ± 0.35	<MDL
**PFHpA**	4.49 ± 0.06	2.98 ± 0.01	4.46 ± 0.04	3.79 ± 0.01	5.26 ± 0.02	3.35 ± 0	7.04 ± 0.07	2.37 ± 1.19	3.63 ± 0.37	3.64 ± 0.09	1.59 ± 0.05	3.82 ± 0.05	5.48 ± 0.06	11.83 ± 0.02
**PFHpS**	<MDL	<MDL	<MDL	<MDL	0.1 ± 0.17	0.15 ± 0.21	0.15 ± 0.25	0.13 ± 0.04	0.04 ± 0.06	<MDL	0.04 ± 0.07	0.07 ± 0.12	0.12 ± 0.17	0.13 ± 0.11
**PFHxA**	11.33 ± 0.02	7.31 ± 0.22	5.47 ± 0.02	6.15 ± 0.03	11.21 ± 0.06	6.25 ± 0.03	17.6 ± 0.05	4.02 ± 1.42	5.89 ± 0.46	5.2 ± 0.07	2.77 ± 0.26	6.85 ± 0.1	10.96 ± 0.06	15.87 ± 2.29
**PFHxS**	1.65 ± 0.03	0.98 ± 0.02	1.03 ± 0.06	0.92 ± 0	3.08 ± 0.09	0.99 ± 0	5.87 ± 0.14	0.76 ± 0.31	0.95 ± 0	0.87 ± 0	0.4 ± 0.06	1.22 ± 0	1.69 ± 0.06	4.42 ± 0.03
**PFNA**	1.19 ± 0.08	1.09 ± 0.07	1 ± 0.04	1.19 ± 0.02	1.36 ± 0.2	1.37 ± 0.43	1.81 ± 0.16	0.69 ± 0.24	0.98 ± 0.15	0.86 ± 0.03	0.49 ± 0.05	1.01 ± 0	1.1 ± 0.01	1.79 ± 0.24
**PFNS**	0.21 ± 0.31	<MDL	1.8 ± 0.53	1.08 ± 0.37	<MDL	2.08 ± 1.61	0.42 ± 0.49	0.03 ± 0.05	0.22 ± 0.27	5.44 ± 8.92	0.08 ± 0.1	0.03 ± 0.01	4.17 ± 5.83	0.13 ± 0.08
**PFOA**	10.67 ± 0.02	10.03 ± 0.22	7.94 ± 0.11	9.27 ± 0.11	12.35 ± 0.36	9.42 ± 0.14	14.78 ± 0.43	4.96 ± 1.68	12.71 ± 4.17	9.01 ± 0.25	5.11 ± 0.32	10.03 ± 0.61	15.65 ± 6.59	16.05 ± 4.98
**PFONS**	0.01 ± 0.01	0.04 ± 0.04	<MDL	<MDL	0.01 ± 0.02	<MDL	0.01 ± 0.02	<MDL	<MDL	0.01 ± 0.02	0.01 ± 0.01	<MDL	<MDL	0.03 ± 0.01
**PFOS**	18 ± 0.77	16.49 ± 4.5	14.64 ± 0.81	16.05 ± 0.59	21.01 ± 2.45	17.46 ± 2.54	34.3 ± 4.92	9.91 ± 4.41	16.26 ± 3.66	12.81 ± 0.72	6.17 ± 1	14.35 ± 0.63	14.79 ± 0.89	19.54 ± 2.75
**PFOUDS**	0.01 ± 0.02	<MDL	0.19 ± 0.17	<MDL	0.01 ± 0.02	<MDL	<MDL	<MDL	<MDL	0.07 ± 0.13	<MDL	0.23 ± 0.2	<MDL	<MDL
**PFPeA**	18.01 ± 0.51	6.55 ± 0.05	7.02 ± 0.12	8.02 ± 0.02	20.72 ± 0.5	7.7 ± 0.17	29.2 ± 0.48	5.19 ± 1.81	5.68 ± 0.03	6.84 ± 0.06	3.72 ± 0.11	7.47 ± 0.22	11.51 ± 1.67	27.02 ± 4.16
**PFPeS**	0.45 ± 0	0.31 ± 0.01	0.25 ± 0	0.37 ± 0.03	0.42 ± 0.01	0.31 ± 0	1.41 ± 0.1	0.2 ± 0.06	0.31 ± 0.17	0.28 ± 0.02	0.1 ± 0	0.45 ± 0.01	0.33 ± 0	0.67 ± 0.03
**PFTeDA**	0.11 ± 0.01	0.04 ± 0.07	0.08 ± 0.02	0.12 ± 0.02	0.13 ± 0.05	0.12 ± 0.17	0.07 ± 0	0.05 ± 0.04	0.1 ± 0.06	0.08 ± 0.05	0.01 ± 0.01	0.13 ± 0.08	0.05 ± 0.02	0.06 ± 0.02
**PFTrDA**	0.08 ± 0.05	0.2 ± 0.19	0.03 ± 0.04	0.1 ± 0.07	0.1 ± 0.01	0.61 ± 0.16	0.13 ± 0.03	0.12 ± 0.11	0.05 ± 0.03	0.14 ± 0.07	0.03 ± 0.02	0.27 ± 0.04	0.04 ± 0.01	0.11 ± 0.04
**PFUdA**	0.31 ± 0.53	3.34 ± 0.85	0.6 ± 1.03	2.09 ± 0.05	0.12 ± 0.11	2.13 ± 0.09	1.55 ± 0.99	<MDL	0.34 ± 0.38	0.81 ± 0.02	0.15 ± 0.03	<MDL	0.43 ± 0.36	0.26 ± 0.22
**∑30PFAS**	194.41 ± 1.99	256.42 ± 29.19	108.07 ± 9.77	166.81 ± 15.74	163.81 ± 66.3	113.66 ± 7.03	216. 52 ± 5.85	76.24 ± 23.61	103.76 ± 8.48	102.66 ± 8.74	46.6 ± 2.8	95.08 ± 1.56	110.96 ± 1.91	211.79 ± 31.34

Note: MDL: method detection limit.

**Table 2:** Concentrations of PBDEs in ng/L at the seven collection points sampled during the dry and wet seasons.

	DRY							WET						
	P1	P2	P3	P4	P5	P6	P7	P1	P2	P3	P4	P5	P6	P7
**BDE-28**	<MDL	<MDL	<MDL	<MDL	<MDL	<MDL	<MDL	0.02 ± 0	0.02 ± 0	0.01 ± 0.01	0.02 ± 0	0.07 ± 0.08	0.03 ± 0.01	0.02 ± 0.01
**BDE-47**	0.02 ± 0	0.02 ± 0	0.36 ± 0.12	0.01 ± 0	0.03 ± 0	0.12 ± 0	0.06 ± 0.01	0.19 ± 0.02	0.14 ± 0	0.24 ± 0.03	0.16 ± 0.01	0.52 ± 0.01	0.52 ± 0.01	0.51 ± 0.01
**BDE-99**	0.02 ± 0	0.02 ± 0	0.54 ± 0.2	0.01 ± 0	<MDL	<MDL	0.01 ± 0	0.01 ± 0	0.21 ± 0.02	0.01 ± 0	0.01 ± 0	<MDL	<MDL	<MDL
**BDE-100**	0.01 ± 0	0 ± 0	0.04 ± 0	0.01 ± 0	0.01 ± 0	0.07 ± 0	0.02 ± 0	0.03 ± 0.01	0.12 ± 0.02	0.02 ± 0.01	0.06 ± 0.02	0.05 ± 0.01	0.16 ± 0.03	0.03 ± 0.01
**BDE-153**	<MDL	<MDL	<MDL	0.01 ± 0	<MDL	<MDL	<MDL	0.01 ± 0	<MDL	0.01 ± 0	<MDL	<MDL	0.01 ± 0	0.01 ± 0
**BDE-154**	<MDL	<MDL	0.01 ± 0	<MDL	0.01 ± 0	0.01 ± 0	<MDL	<MDL	<MDL	<MDL	0.01 ± 0	<MDL	0.01 ± 0	0.01 ± 0
**BDE-183**	0.6 ± 0.08	0.81 ± 0.06	0.8 ± 0.18	0.02 ± 0	0.03 ± 0	0.03 ± 0	0.03 ± 0	0.18 ± 0.02	0.07 ± 0.01	0.25 ± 0.02	<MDL	<MDL	0.02 ± 0	<MDL
**BDE-209**	0.34 ± 0.04	0.34 ± 0.05	0.8 ± 0.04	0.69 ± 0.07	0.45 ± 0.16	0.68 ± 0.2	1.89 ± 0.03	2.48 ± 0.28	1.93 ± 0.03	2.43 ± 0.37	6.9 ± 1.12	6.35 ± 1.62	4.09 ± 0.39	2.86 ± 0.7
**∑8BDEs**	1 ± 0.06	1.21 ± 0.11	2.56 ± 0.31	0.74 ± 0.07	0.55 ± 0.16	0.92 ± 0.21	2.02 ± 0.02	2.92 ± 0.27	2.51 ± 0.02	2.97 ± 0.39	7.15 ± 1.12	6.99 ± 1.55	4.84 ± 0.4	3.44 ± 0.7

Note: MDL: method detection limit.

## Data Availability

The data that support the findings of this study are available from the corresponding author [JSA] upon reasonable request.

## Supplementary Materials

The following supporting information can be downloaded at: https://www.mdpi.com/article/10.3390/toxics14020148/s1, Table S1. Target PBDEs and the corresponding Internal standards. Table S2. List of 30 PFAS with their internal standards applied for quantitation and list of 24 PFAS in the secondary standard. Table S3. The sampling points data. Table S4. The LC gradient conditions. Table S5. MS parameters for LC-MS/MS analysis. Table S6. Summary of the Multiple Reaction Monitoring (MRM) method for the PFAS analysis, including compounds, precursor and product ions, retention time (RT; in minutes), delta RT, fragmentor, collision energy, and cell accelerator voltage. Table S7. Retention time (RT) and m/z ions selected for PBDEs target analytes. Table S8. Method validation results for PFAS SPE method (MDL) and instrument detection limit (IDL) of PFAS and method validation of direct injection method described by Li et al. [47]. Table S9. PBDE limits of detection (MLD) and quantification (MLQ).
