# Supplementary material for: Assessment of Per- and Poly-Fluoroalkyl Substances (PFAS) and Polybrominated Diphenyl Ethers (PBDEs) in Surface Waters Used for Urban Water Supply in Brazil
Source: Toxics. 2026 Feb 2;14(2):148. doi: 10.3390/toxics14020148 (PMC12945003; doi:10.3390/toxics14020148)
Supplement: Supplementary file 1 [file toxics-14-00148-s001.zip › toxics-4113260-supplementary.pdf]

Assessment of per- and polyfluoroalkyl substances (PFAS) and Polybrominated diphenyl ethers (PBDEs) in surface waters in urban water supply from Brazil

Juliana de Souza-Araujo[1*], Isadhora Camargo dos Santos[1], Hansel David Burgos Melo[1], Leila Soledade Lemos[2], Natalia Quinete[2*] & André Henrique Rosa[1*]

[1] Instituto de Ciência e Tecnologia, UNESP – Campos Sorocaba.

[2] Institute of Environment, Department of Chemistry and Biochemistry, Florida International University, 3000 NE151st street, Biscayne Bay Campus, North Miami, FL33181, USA

***Corresponding authors:**

j.araujo.bio@gmail.com

andre.rosa@unesp.br

nsoaresq@fiu.edu

Table S1: Target PBDEs and the corresponding Internal standards

PBDEs	Compound Name	Internal Standard (IS)	Recovery Determination Standard (RDS)
BDE 28	2,4,4′-Tribromodiphenyl ether	BDE-77	$^{13}C_{12}$ – BDE - 100
BDE 47	2,2′,4,4′-Tetrabromodiphenyl ether		
BDE 99	2,2′,4,4′,5-Pentabromodiphenyl ether		
BDE 100	2,2′,4,4′,6-Pentabromodiphenyl ether		
BDE 153	2,2′,4,4′,5,5′-Hexabromodiphenyl ether	BDE-128	
BDE 154	2,2′,4,4′,5,6′-Hexabromodiphenyl ether		
BDE 183	2,2′,3,4,4′,5′,6-Heptabromodiphenyl ether		
BDE-209	Decabromodiphenyl ether	$^{13}C_{12}$ - BDE 209	

Table S2: List of 30 PFAS with their internal standards applied for quantitation and list of 24 PFAS in the secondary standard.

PFAS	Compound Name	Internal Standard (IS)	Secondary Standard
4-2 FTS	Sodium 1H,1H,2H,2H-perfluoro-1-hexanesulfonate	M2 4-2 FTS	Yes
6-2FTS	Sodium 1H,1H,2H,2H-perfluoro-1-octanesulfonate	M2 6-2FTS	Yes
8-2 FTS	Sodium 1H,1H,2H,2H-perfluoro-1-decanesulfonate	M2 8-2 FTS	Yes
ADONA	Sodium dodecafluoro-3H-4,8-dioxanonanoate	M2 6-2FTS	
FBSA	Perfluoro-1-butanesulfonamide	M GenX	
FHxSA	Perfluoro-1-hexanesulfonamide	M8 PFOA	
FOSA	Perfluoro-1-octanesulfonamide	M8 FOSA	
HFPO-DA	2,3,3,3-Tetrafluoro-2-(1,1,2,2,3,3,3-heptafluoropropoxy)-propanoic acid	M GenX	

N-EtFOSAA	N-ethylperfluoro-1-octanesulfonamidoacetic acid	d5 N-EtFOSAA	Yes
N-MeFOSAA	N-methylperfluoro-1-octanesulfonamidoacetic acid	d3 N-MeFOSAA	Yes
PFBA	Perfluoro-n-butanoic acid	MPFBA	Yes
PFBS	Potassium perfluoro-1-butanesulfonate	M3 PFBS	Yes
PFDA	Perfluoro-n-decanoic acid	M6 PFDA	Yes
PFDoA	Perfluoro-n-dodecanoic acid	M PFDoA	Yes
PFDS	Sodium perfluoro-1-decanesulfonate	M7 PFUdA	Yes
PFHpA	Perfluoro-n-heptanoic acid	M4 PFHpA	Yes
PFHpS	Sodium perfluoro-1-heptanesulfonate	M3 PFHxS	Yes
PFHxA	Perfluoro-n-hexanoic acid	M5 PFHxA	Yes
PFHxS	Potassium perfluorohexanesulfonate	M3 PFHxS	Yes
PFNA	Perfluoro-n-nonanoic acid	M3 PFHxS	Yes
PFNS	Sodium perfluoro-1-nonanesulfonate	M8 PFOS	Yes
PFOA	Perfluoro-n-octanoic acid	M8 PFOA	Yes
PFONS	Potassium 9-chlorohexadecafluoro-3-oxanonane-1-sulfonate	M8 PFOS	
PFOS	Potassium perfluorooctanesulfonate	M8 PFOS	Yes

PFOUdS	Potassium 11-chloroeicosafluoro-3-oxaundecane-1-sulfonate	M PFDoA	
PFPeA	Perfluoro-n-pentanoic acid	M5 PFPeA	Yes
PFPeS	Sodium perfluoro-1-pentanesulfonate Potassium	M GenX	Yes
PFTeDA	Perfluoro-n-tetradecanoic acid	M2 PFTeDA	Yes
PFTrDA	Perfluoro-n-tridecanoic acid	M2 PFTeDA	Yes
PFUdA	Perfluoro-n-undecanoic acid	M7 PFUdA	Yes

Table S3. The sampling points data.

Sampling point	Place	Reference	Latitude	Longitude
P1	Itupararanga	Headwaters of the Sorocamirim and Sorocabuçu rivers	-23.619973	-47.232861
P2	Itupararanga	In front of a resort	-23.627814	-47.318018
P3	Itupararanga	In front of a residential building	-23.659237	-47.357053
P4	Itupararanga	Near the dam (woods/pasture)	-23.620991	-47.392623
P5	Sorocaba river	Upstream of Sorocaba city	-23.528127	-47.447807
P6	Sorocaba river	Center of Sorocaba city	-23.468122	-47.440855
P7	Sorocaba river	Downstream of Sorocaba city	-23.43579	-47.516349

Table S4. The LC gradient conditions.

Time (min)	A [%]	B [%]	Flow (ml/min)
0.00	90	10	0.4

8.00	5	95	0.4
11.00	5	95	0.4
12.00	90	10	0.4

A=5mM Ammonium formate and B=Methanol

Table S5. MS parameters for LC-MS/MS analysis

Parameter	Setting
MS Acquisition	Dynamic MRM
Cycle Time	500 ms
Ion Source	ESI negative
Drying Gas Temperature & Flow	150 ℃ & 10 L/min
Nebulizer	15 psi
Sheath GasTemperature & Flow	300 ℃ & 10 L/min
Capillary	2000 V
Nozzle Voltage	0 V

Table S6. Summary of the Multiple Reaction Monitoring (MRM) method for the PFAS analysis, including compounds, precursor and product ions, retention time (RT; in minutes), delta RT, fragmentor, collision energy, and cell accelerator voltage.

Compound Name	Precursor Ion	Product Ions	Retention Time (min)	Delta Retention Time	Fragmentor	Collision Energy	Cell Accelerator Voltage
4-2 FTS	327	80.9	5.69	3	125	32	5
6-2FTS	427	406.8; 79.9	6.95	3	125	24	5
8-2 FTS	527	506.8; 80.9	7.65	3	170	28	5
Adona	377	250.9	6.54	3	70	10	5
d3 N-MeFOSAA	573	419	8.1	3	115	20	5
d5 N-EtFOSAA	589	419	8.18	3	115	20	5
FBSA	298	77.9	6.83	3	100	28	5
FHxSA	398	77.9	7.95	3	100	28	5
FOSA	497.9	77.9	8.63	3	125	36	5
GenX	285	169	5.88	3	108	12	5
M GenX	287	185	5.88	3	108	20	5
M PFDoA	615	570	8.21	3	79	8	5

M2 4-2 FTS	329	81	5.66	3	125	28	5
M2 6-2FTS	429	81	6.94	3	125	32	5
M2 8-2 FTS	529	81	7.66	3	170	36	5
M2 PFTeDA	715	670	8.72	5	100	13	5
M3 PFBS	302	99	4.99	3	100	33	5
M3 PFHxS	402	99	6.57	3	100	41	5
M4 PFHpA	367	322	6.52	3	72	8	5
M5 PFHxA	318	273	5.79	3	70	8	5
M5 PFPeA	268	223	4.35	3	60	4	5
M6 PFDA	519	474	7.7	3	81	8	5
M7 PFUdA	570	525	7.98	3	73	8	5
M8 FOSA	506	78	8.63	3	125	36	5
M8 PFOA	421	376	7.01	3	69	8	5
M8 PFOS	507	99	7.37	3	100	46	5
M9 PFNA	472	427	7.39	3	66	8	5
MPFBA	217	172	2.18	3	60	8	5
N-EtFOSAA	584	525.9; 418.9	8.18	3	115	20	5
N-MeFOSAA	570	482.9;418.9	8.09	3	115	16	5
PFBA	213	168.9	2.18	3	60	8	5
PFBS	298.9	98.9;80	4.98	3	100	33	5
PFDA	513	469	7.7	3	81	8	5
PFDoA	613	569; 268.7	8.21	3	79	8	5
PFDS	598.9	99	7.93	3	100	56	5
PFDS	598.9	80	7.93	3	100	88	5

PFHpA	362.9	319; 169	6.52	3	72	5	5
PFHpS	448.9	98.7; 79.7	7.01	3	100	44	5
PFHxA	313	268.9	5.79	3	70	4	5
PFHxS	398.9	99;80	6.56	3	100	41	5
PFNA	463	419;169	7.39	3	66	8	5
PFNS	548.9	98.9; 79.9	7.67	3	165	48	5
PFOA	413	369;169	7.01	3	69	8	5
PFONS	530.9	350.9	7.44	3	100	17	5
PFOS	498.9	99; 80	7.37	3	100	46	5
PFOUDS	630.9	450.9	7.97	3	100	17	5
PFPeA	263	218.9	4.3	3	60	4	5
PFPeS	348.9	98.9;79.9	5.97	3	135	36	5
PFTeDA	713	669;169	8.6	3	100	13	5
PFTrDA	663	619;169	8.41	3	91	9	5
PFUdA	563	519	7.97	3	73	8	5
4-2 FTS	327	306.9	5.69	3	125	20	5

Table S7: Retention time (RT) and m/z ions selected for PBDEs target analytes

Congeners	Retention Time (min)	Ions	
BDE 77	16.00	485.7	483.7
BDE 28	14.35	407.8	405.7
BDE 47	15.58	485.7	325.8
BDE 99	16.53	563.6	403.8
$^{13}C_{12}$ – BDE - 100	16.31	575.7	578.0
BDE 100	16.31	403.8	405.7
BDE 153	17.35	483.7	485.7
BDE 154	17.05	483.7	485.7
BDE 183	18.04	563.6	561.6
$^{13}C_{12}$ - BDE 209	24.18	811.6	809.6
BDE 209	24.18	799.6	801.6

Table S8: Method validation results for PFAS SPE method (MDL) and instrument detection limit (IDL) of PFAS and method validation of direct injection method described by Li et al.(2021).

**Compounds**	**MDL (ng/L)**	**MDL Intraday RSD**	**MDL Interday RSD**	**MDL Recovery (%)**	**Matrix Effect (tap water)**	**Matrix Effect (Surface water)**	**IDL (ng/L)**	**IDL Intraday RSD**	**IDL Interday RSD**	**IDL Recovery (%)**	**Matrix Effect**
PFBA	0.05	4.10	5.36	102	-10.8	-6.24	0.26	6.01	5.19	98.7	-1.25
FBSA	0.05	2.28	5.93	113	-12.7	3.43	2.14	9.83	10.7	105	4.00
PFBS	0.01	0.80	1.27	101	-23.6	4.18	0.99	8.83	6.45	106	7.96
PFPeA	0.13	6.10	10.10	103	6.45	13.02	9.00	7.73	9.21	103	4.31
PFPeS	0.02	1.38	1.38	102	2.33	15.8	0.51	6.69	7.13	97.7	4.15
PFHxA	0.01	3.59	3.73	98.9	10.8	3.44	1.93	4.05	4.08	102	3.48
FHxSA	0.03	3.77	6.23	91.0	-25.6	-53.8	3.47	6.08	15.3	71.9	15.6
PFHxS	0.04	4.67	3.55	102	-28.7	3.43	2.31	11.4	11.6	98.8	1.28
4-2 FTS	0.03	2.68	1.96	103	2.97	4.53	5.93	8.27	9.47	103	3.19
Adona	0.02	4.67	4.66	100	-49.3	-21.3	0.98	13.3	6.56	93.9	11.1
GenX	0.02	1.43	2.99	100	-9.60	-7.39	5.90	7.09	5.45	100	1.64
PFHpA	0.05	3.62	10.00	121	7.83	-45.3	1.76	5.97	5.76	105	7.19
PFHpS	0.04	3.95	4.04	102	7.19	11.0	8.55	10.1	7.44	103	3.57
PFOA	0.04	6.18	9.29	98.9	-17.3	-10.2	2.04	5.11	7.23	102	5.57
FOSA	0.04	4.34	2.99	106	-14.6	0.61	5.16	14.7	19.7	67.0	3.18
PFOS	0.04	4.23	13	103	-18.4	-16.6	1.35	8.85	11.9	96.8	6.32
6-2 FTS	0.35	3.47	7.04	128	15.3	-21.5	44.6	14.45	23.1	98.3	-10.9
PFONS	0.02	2.9	4.68	92.5	10.6	13.8	1.97	17.5	21.9	93.8	3.85
PFNA	0.03	4.1	5.79	91.6	-2.05	14.5	4.66	6.75	6.08	97.5	4.27
N-MeFOSAA	0.29	7.55	8.29	90.3	-17.0	2.75	97.3	24.3	25.9	88.1	1.22

N-EtFOSAA	0.45	9.61	4.45	94.5	12.9	9.80	54.2	19.2	19.0	83.8	-2.73
PFNS	0.02	9.68	12.1	64.7	7.82	3.04	2.77	16.7	19.2	80.2	-9.27
PFDA	0.02	2.76	5.08	98.9	11.8	15.8	3.58	14.3	14.9	90.1	4.08
PFDS	0.33	9.74	14.5	98.6	0.81	-6.91	18.3	24.0	21.0	101	-5.64
8-2 FTS	0.34	3.25	7.15	94	1.36	26.0	41.6	14.9	15.4	82.2	10.1
PFUdA	0.20	3.69	5.22	105	25.5	17.6	7.98	12.1	15.0	98.1	28.9
PFDoA	0.22	3.42	6.83	113	-14.9	-7.49	32.7	11.9	10.8	87.6	5.88
PFTrDA	1.37	4.82	7.25	99.8	-6.59	-8.35	107	25.3	27.8	91.8	-57.0
PFTeDA	1.99	6.55	20.2	128	10.2	-4.1	205	19.6	38.1	87.1	-13.2
PFONDS	0.36	9.71	13.3	103.8	-54.1	0.94	66.0	16.7	11.7	117	-3.68

RSD: relative standard deviation.

Table S9: PBDE limits of detection (MLD) and quantification (MLQ)

Compound	MLD (pg/µL)	MLQ (pg/µL)
BDE-28	1.99	5.7
BDE-47	1.83	5.2
BDE-99	3.9	11.1
BDE-100	1.99	5.7
BDE-153	2.48	6.3
BDE-154	2.32	5.9
BDE-183	2.88	7.3
BDE-209	105.88	338.0
